# Periprosthetic joint infections after total hip replacement: an algorithmic approach

**DOI:** 10.1051/sicotj/2019004

**Published:** 2019-02-28

**Authors:** Mohamed Sukeik, Fares Sami Haddad

**Affiliations:** 1 Foothills Medical Centre 1403 29 St NW Calgary AB T2N 2T9 Canada; 2 University College London Hospital 235 Euston Road London NW1 2BU UK

**Keywords:** Periprosthetic, Joint, Infection, Hip replacement, Arthroplasty

## Abstract

An algorithm for managing periprosthetic joint infections (PJIs) after total hip replacement (THR) surgery using a multidisciplinary approach and a clearly defined protocol may improve infection eradication rates. In this article, we present an algorithm for the management of different types of PJIs including the acutely infected cemented and cementless THRs where the components are well-fixed postoperatively and when the infection is secondary to haematogenous spread in previously well-functioning and well-fixed implants. For chronic PJIs where the components are often loose, the standard treatment includes a two-stage revision procedure. However, in a highly selected subset of patients, a single-stage approach has been utilised with high rates of eradicating infections.

## Introduction

Health services are experiencing an exponential global rise in numbers of lower limb arthroplasty procedures performed for an ageing population. Over the last five years, the UK National Health Service witnessed a growth of hip and knee arthroplasty procedures by 4000–5000 cases/year [1]. Subsequently, even a minimal prosthetic joint infection (PJI) rate of 0.57% constitutes a major concern [2], especially with the financial burden of a single revision procedure for sepsis exceeding £21,000 [3]. The picture is further complicated by the continuous metamorphosis and emergence of new resistant bacterial strains as well as infections with rare organisms [4].

Challenges including diagnostic uncertainty, immunocompromised patients, recurrent infection, infection around a well-fixed implant and substantial bone loss require careful preoperative assessment and well-defined treatment plans [5]. However, there is still no consensus over a standard treatment strategy for PJIs which has accounted for the extensive variability in infection eradication rates in the literature [6,7]. Therefore, an algorithm utilising a multidisciplinary approach and a clearly defined protocol may improve infection rates and contribute to standardising management of PJI after THA.

We present in this study an algorithmic approach to treating different types of PJIs after THA surgery. The protocol involves aggressive surgery removing all mobile and non ingrown parts and exchanging them at the same sitting for acute infection, and selective single- versus two-stage strategy for established infections based on host, organism and local factors.

## Protocol

In a case of suspected THA infection, the patient should be promptly referred to the specialist hip team which utilises a multi-disciplinary approach in managing such infections as this is a specialised procedure and there is no role for simple incision and drainage or repetitive washouts which result in emergence of resistant microorganisms [5,8]. Clinical presentation (pain, fever, swelling, skin redness, discharging sinus), serologic testing (erythrocyte sedimentation rate [ESR] > 30 mm/h; C-reactive protein [CRP] > 10 mg/L), hip aspiration and biopsy with microbiology and cell count analyses help us diagnose PJI [8–10]. Definitive diagnosis however, is established when three-to-six specimens are sampled from different sites at the time of surgery (e.g., capsule, femur and acetabulum) and the same microorganism is cultured from at least two specimens [10–13]. The extent of infection and the interval for which it has been present play a role in the choice of treatment and the chances for successful eradication of infection as follows:

### 1) Acute infection

This is defined as an infection occurring within 6–8 weeks of the index operation (primary or revision) or of haematogenous spread from a confirmed source of infection elsewhere in a previously well-functioning implant [12,14,15]. In haematogenous infections, a full workup to establish the source of infection should be undertaken preoperatively, including a comprehensive history of recent systemic infections or invasive procedures causing bacteremic seeding of the hip, and investigations should include a throat swab, chest radiograph, and urine, stool and blood cultures [14]. Decision to perform surgery should be based on a high index of suspicion from clinical presentation and serologic testing. Diagnostic aspiration and biopsies in acute infections may delay surgical intervention and also carries variable sensitivity and specificity rates for diagnosing infection 0.50–0.93 and 0.82–0.97, respectively [16]. Treatment of acute infection is subdivided according to the type of prosthetic fixation of the original implant:

#### a) Cemented prostheses

An aggressive open debridement with exchange of mobile parts and retention of the implant in stable components with no evidence of immunosuppression, and overlying soft tissue and skin of good condition is associated with good results of infection control, especially when the infection is diagnosed within the first week after the index procedure [13–15,17,18]. The aim of rapid intervention with thorough open debridement is to prevent the production of any biofilm by the infecting organism, paramount for successful treatment of infection [7,19]. Patients undergo an open complete synovectomy, multiple tissue sampling, exchange of femoral heads and acetabular inserts, debridement of all aspects of the joint, irrigation with solutions such as hydrogen peroxide and Betadine^®^ solutions, and pulsatile lavage [15,18,20].

#### b) Cementless prostheses

For acute haematogenous infections in previously well-functioning and well-fixed cementless implants, the same protocol for cemented prostheses can be followed as detailed above. However, in acute postoperative infections, once the debridement is complete and samples are sent, another option is to proceed to a single-stage revision procedure where all drapes, gowns, gloves and equipment are changed to create a new, sterile environment. A direct exchange single-stage cementless THA can then be performed as this represents an ideal opportunity to remove both the implant and its biofilm prior to ingrowth [19,21]. Hansen et al. [21] published their series of 27 patients who were treated using this strategy and at a minimum of 27 months reported that 19 of the 27 patients (70%) retained their implants but four out of those patients required further debridement in order to obtain control of infection.

For both treatment modalities, patients need to continue antibiotic therapy tailored to the sensitivities of intraoperative cultures for at least six weeks until inflammatory markers (CRP, ESR) and the plasma albumin concentration return to within normal limits [8,14]. Early conversion to oral antibiotics is dictated by sensitivities and consultation with the microbiology team with whom multidisciplinary meetings should be held on a regular basis [18,20].

### 2) Chronic infection

In chronic PJIs, the protocol includes careful assessment of local soft tissues, baseline CRP and ESR, and hip aspiration combined with tissue biopsy as this has shown improved sensitivity and accuracy for diagnosing infection after at least four weeks of discontinuing any antibiotic therapy [18,22]. Plain anteroposterior and lateral radiographs should also be performed, with additional CT if deemed necessary for further acetabular assessment [8,23,24]. Once the diagnosis of PJI is suggested by clinical findings and investigations, and the patients are deemed fit and are agreeable to having surgery, patients are considered for either a single- or two-stage revision procedure according to the following:

#### a) Single-stage revision

A single-stage revision is carried out under strict conditions including: minimal/moderate bone loss, non-immunocompromised patients, healthy soft tissues, a known organism with known sensitivities, and when appropriate antibiotics are available [23,25–27]. The operation is split into two parts; the first consists of an open aggressive debridement with removal of all components and cement, during which multiple samples are sent to microbiology and irrigation with hydrogen peroxide and Betadine^®^ solutions, and then pulsatile lavage is done. The area is then soaked in aqueous betadine and the wound edges approximated. This is considered to be the end of the first part of the operation and the patient is re-draped and new instruments are used. The surgical team rescrub and put on new gowns [23]. After a further lavage, implantation of a new prosthesis is performed using antibiotic-loaded cement (ALC) or antibiotic-loaded bone graft as needed [18,26,27]. Patients continue antibiotic therapy tailored to the sensitivities of intraoperative cultures for at least six weeks until inflammatory markers (CRP, ESR) and the plasma albumin concentration return to within normal limits. The change from intravenous to oral therapy is effected as soon as full organism sensitivity profile is available [28,29].

#### b) Two-stage revision

This is the gold standard for treatment of chronically infected and complex THA infections as the successful eradication of a PJI is over 90% [24,30–32]. Intraoperatively, the first part of the operation is similar to a single-stage revision. However, after rescrubbing and re-draping, a temporary articulating ALC spacer is implanted instead. This spacer normally contains broad spectrum antibiotics such as vancomycin and gentamicin to cover organisms commonly encountered with deep periprosthetic infections whilst reducing the development of resistant strains [18,33]. Postoperatively, the patient is allowed to mobilise partial weight-bearing with crutches and is discharged home when deemed safe. Antibiotic therapy tailored to the sensitivities of intraoperative cultures is continued for 4–6 weeks [18,24,29]. The decision to proceed with insertion of a new prosthesis is determined by the clinical response of the patient including wound healing, inflammatory and nutritional markers indicating resolution of infection together with performing a further aspiration which is negative [8,18,24]. At the second stage, the spacer is removed and the underlying cement mantle is fragmented and removed piecemeal, without sacrificing bone stock. Appropriate implants are then reimplanted with either cemented or cementless components, and allografts may be used in cases of severe bone loss. The importance of a multi-disciplinary team approach as well as strict adherence to the above protocol is important in order to achieve high rates of infection control [25,29,34].

Regardless of the treatment strategy followed, all patients should be followed up postoperatively at two and six weeks, six months, one year, and then on a yearly basis, looking for clinical symptoms and signs of infection, as well as CRP and ESR level testing. Plain radiographs including an AP pelvis and lateral of both hips should be requested at every follow-up appointment. Stem position, radiolucencies and osteolysis should be assessed. The stem angle is classified as neutral, varus or valgus. A stem angle is considered neutral if its axis is within 2° of the femoral shaft axis. Femoral and acetabular radiolucencies are classified according to Gruen et al. [35] and DeLee and Charnley [36] zones, respectively. Loosening is diagnosed if the radiolucent zone around one or both components is 2 mm or more in width and a patient has symptoms on weightbearing and motion that are relieved by rest [37]. Osteolytic lesions are documented and classified on the basis of their size (linear or expansile) and their location according to previously published criteria by Zicat et al. [38]. Of note, though, is that substantial interobserver variability can be expected using these systems [39,40]. Definition of eradication of infection has been variable in the literature, but mostly includes the absence of clinical, serologic, and radiographic signs of infection and absence of death secondary to infection or treatment during the follow-up period. Failure on the other hand includes any major operation performed in any subgroup of patients for eradication of infection, including a two-stage revision, excision arthroplasty, arthrodesis and amputation, or the need for long-term antibiotic suppression. A reinfection is considered to be an infection with the same or another organism.

## Discussion

Despite the relatively low rates of PJIs after THAs, they remain a leading cause of revision surgery due to an ever-increasing number of hip arthroplasties performed yearly for an ageing population [8]. Difficulties with reaching a consensus on what defines infection and which strategy best eradicates it led to extensive variability in infection rates in the literature, until recent efforts from the International consensus meeting on managing PJIs defined what constitutes an infection [12]. Specialist tertiary centres dealing with PJIs on a regular basis may improve infection-free survival and contribute to a global approach for managing PJI. Therefore, we aimed in this study at presenting our preferred algorithm for treating PJIs after THA surgery.

Results for eradication of infection using an aggressive early debridement and exchange of mobile parts for acute infections as detailed here and two-stage revision for chronic infections where a clear protocol has been followed yield high rates of eradicating PJIs (Table 1) [41–43]. It is of note, though, that the inclusion and exclusion criteria, as well as management protocols, varied among studies reported in the literature, occasionally including all types of periprosthetic infections rather than acute or chronic infections only. Additionally, some of the studies did not differentiate between hips and knees when reporting their findings which resulted in a wide range of eradicating infection (Table 1).

**Table 1:** Previous studies reporting PJI eradication rates following various treatment strategies.

Author	Treatment	Infection site	Number of cases	Exchange of mobile parts	Eradication rate (%)	Follow-up in years
Aboltins et al. [48]	Debridement	Hip/Knee	17	Yes	88.2	2.3
Klouche et al. [57]	Debridement	Hip	12	Partly	75	3.3
Krasin et al. [60]	Debridement	Hip	7	No	71	2.5
Martinez-Pastor et al. [56]	Debridement	Hip/Knee	47	Yes	74.5	1.2
Sukeik et al. [14]	Debridement	Hip	26	Yes	77	6.6
Choi et al. [46]	Single	Hip	17	–	82	5.2
Klouche et al. [57]	Single	Hip	38	–	100	2
Winkler et al. [45]	Single	Hip	37	–	92	4.4
Zeller et al. [58]	Single	Hip	157	–	95	5
Oussedik et al. [54]	Single	Hip	11	–	100	6.8
Berend et al. [30]	Two-stage	Hip	186	–	83	4.4
Klouche et al. [57]	Two-stage	Hip	46	–	98	>2
Masri et al. [59]	Two-stage	Hip	29	–	90	>2
Ibrahim et al. [34]	Two-stage	Hip	125	–	96	>5

On the other hand, single-stage revisions for chronic infections are regaining momentum, and studies reporting high rates of eradicating infection certainly reflect a strict protocol followed similar to the one described in this review [18,44,45]. Single-stage direct exchange protocol for acutely infected cementless THAs remains a novel approach which has not yet gained popularity, but presents a time-limited opportunity to remove the implants prior to ingrowth in a cementless THA [21]. In comparison with aggressive debridement with exchange of mobile parts in cemented THAs [14], it showed almost similar results for eradication of infection (70% vs. 77%) with a single operation, whereas a few of the cases in the debridement group required several wash outs with the additional soft tissue trauma caused before eradication of infection [21].

A number of challenging cases can add to the complexity of dealing with PJIs. For example, culture negative infections, resistant organisms, reconstruction of large bony defects after removal of autologous or allogeneic bone grafts used in primary operations for hip dysplasia or revision surgery and dealing with metal on metal bearings and dual mobility implants. Principles for treating such difficult cases remain the same but with special considerations for each case. For example, in culture negative and resistant organisms, diagnosis can be challenging, but once established using all the diagnostic tools available and applying the International Consensus Meeting (ICM) on management of PJIs diagnostic criteria [12], treatment strategies should follow a two-stage reimplantation procedure as debridement and exchange of mobile parts and single-stage revisions are associated with higher failure rates [18,46–49]. Patients who fail treatment may require salvage procedures such as long-term antibiotics, resection arthroplasty, fusion and amputation [50].

Patients who had undergone autologous or allogeneic bone grafting as part of their primary operations for hip dysplasia or revision surgery constitute another challenging group of patients. As there is limited evidence in the literature guiding treatment of these groups of patients, it is our preference that if such bone grafts have osseointegrated and it has been a number of years since the primary operation with no evidence of an underlying chronic infection that there is no need to remove those grafts. Otherwise, treatment for the different types of PJIs encountered remains the same as detailed in the above protocol. We also prefer removing any underlying metalwork from the primary procedure including screws utilised for the original fixation of the cup if it is safe and feasible to do so, as there is evidence that retained metalwork may contribute to incomplete debridement and possible recurrence of the infection [18,20].

Metal on metal bearings constitute another challenge due to the difficulty of establishing the correct diagnostic thresholds for inflammatory markers in the serum and synovial fluid samples. In fact, a number of studies suggested that CRP and ESR are not reliable alone in diagnosing PJI in metal on metal cases and that the synovial fluid WBC count can frequently be falsely positive and, therefore, should be relied on only if a manual count is done and a differential can be performed [12,51,52]. The ICM also suggests being cautious whilst applying its diagnostic criteria for PJIs in metal on metal cases for the same reasons [12].

Dual mobility bearings have been associated with lower dislocation rates and no increase in complications when compared with other THA bearings. Infections in particular are not higher with dual mobility bearings. Therefore, management of PJIs in this context has not been widely discussed in the literature. However, in the few published articles a number of points should be noted including the fact that diagnosis of PJI can be challenging, especially in the setting of intraprosthetic dislocation due to associated metal debris, and the principles should follow the above recommendations in those cases [12,18]. In the cases of acute infection, shell stability should be assessed intraoperatively as it is easy to revise it in the early postoperative phase [53]. Additionally, dual mobility bearings allow easier exchange of the liner, but the head requires removal which may risk damage to the taper. In chronic cases, a two-stage revision procedure remains the gold standard. However, single-stage revision may still be considered in a strictly selected group of patients as detailed previously [53].

## Conclusion

In conclusion, we present a clear protocol for treating periprosthetic hip arthroplasty infections which has been supported by a number of studies in the literature from centres dealing with PJIs on a regular basis, yielding high rates of eradicating infection. We also agree that only through the use of standardised terminology that an international language of comparative results will be feasible and, therefore, we support efforts made to standardise the definition of PJI [10,13,54,55]. However, in view of the heterogeneity of clinical presentation and variability of diagnostic tests’ validity and reliability in diagnosing infection, the debate for a common strategy of treatment has yet to be finalised.

## Conflict of interest

Authors certify that they have no financial conflict of interest.
